# A Broad-Host-Range Plasmid Outbreak: Dynamics of IncL/M Plasmids Transferring Carbapenemase Genes

**DOI:** 10.3390/antibiotics11111641

**Published:** 2022-11-17

**Authors:** María Getino, María López-Díaz, Nicholas Ellaby, John Clark, Matthew J. Ellington, Roberto M. La Ragione

**Affiliations:** 1 Department of Comparative Biomedical Sciences, School of Veterinary Medicine, University of Surrey, Guildford GU2 7AL, UK; 2 UK Health Security Agency, Colindale, London NW9 5EQ, UK; 3 Department of Microbiology, Epsom and St Helier University Hospitals NHS Trust, Carshalton SM5 1AA, UK; 4 School of Biosciences and Medicine, University of Surrey, Guildford GU2 7XH, UK

**Keywords:** antimicrobial resistance, carbapenemase, Enterobacterales, plasmid, conjugation, IncL/M, NDM-1, OXA-48

## Abstract

IncL/M broad-host-range conjugative plasmids are involved in the global spread of *bla*_OXA-48_ and the emergence of *bla*_NDM-1_. The aim of this study was to evaluate the transmission potential of plasmids encoding the emergent NDM-1 carbapenemase compared to the pandemic OXA-48. The conjugation rate and fitness cost of IncM2 and IncL plasmids encoding these carbapenemase genes were tested using a variety of host bacteria. Genomic analysis of uropathogenic *Escherichia coli* SAP1756 revealed that *bla*_NDM-1_ was encoded on an IncM2 plasmid, which also harboured *bla*_TEM-1_, *ble*_MBL_ and *sul1* and was highly similar to plasmids isolated from the same geographical area. Conjugation experiments demonstrated that NDM-1 and OXA-48-carrying plasmids transfer successfully between different Enterobacterales species, both in vitro and in vivo. Interestingly, *E. coli* isolates tested as recipients belonging to phylogroups A, B1, D and F were able to receive IncM2 plasmid pSAP1756, while phylogroups B2, C, E and G were not permissive to its acquisition. In general, the IncL OXA-48-carrying plasmids tested transferred at higher rates than IncM2 harbouring NDM-1 and imposed a lower burden to their host, possibly due to the inactivation of the *tir* fertility inhibition gene and reflecting their worldwide dissemination. IncM2 plasmids carrying *bla*_NDM-1_ are considered emergent threats that need continuous monitoring. In addition to sequencing efforts, phenotypic analysis of conjugation rates and fitness cost are effective methods for estimating the pandemic potential of antimicrobial resistance plasmids.

## 1. Introduction

Carbapenemase-producing Enterobacterales (CPE) are classified as critical priority pathogens by the World Health Organization (WHO) [1]. Carbapenemases are acquired β-lactamases that prevent carbapenems, antibiotics used in healthcare settings as last resort, from effectively clearing bacterial infections caused by CPE. Apart from carbapenems, these enzymes are capable of hydrolysing penicillins, cephalosporins and monobactams. They are classified in class A, B or D, depending on the composition of their active site (serine for classes A and D and divalent cations for class B) [2]. Class B β-lactamases, including metallo-β-lactamases, hydrolyse a broad variety of β-lactams (except monobactams) in a zinc-dependent manner, while Class D β-lactamases, including OXA-48-like carbapenemases, produce a relatively weak hydrolysis of penicillins and carbapenems, but not cephalosporins, which makes them more difficult to detect [3]. These enzymes are frequently encoded on mobile genetic elements such as conjugative plasmids along with numerous other antimicrobial resistance (AMR) genes [4].

Conjugative plasmids are considered major vehicles in the spread of AMR [5]. They are capable of horizontally transferring between bacteria, ensuring their stability through toxin-antitoxin and partitioning systems, as well as counteracting their own fitness cost via mutation. These mechanisms allow conjugative plasmids to successfully spread and persist, even in the absence of antibiotics. Broad-host-range conjugative plasmids are of special high risk as they enable the transfer of multiple AMR genes between different bacterial species. 

An example of broad-host-range plasmids involved in the spread of AMR genes is the IncL/M incompatibility group, responsible for the worldwide dissemination of the *bla*_OXA-48_ carbapenemase gene [6]. OXA-48 is a class D β-lactamase first isolated from a *K. pneumoniae* resistant to all β-lactams in Turkey in 2001 [7]. The *bla*_OXA-48_ gene, frequently carried by a conserved and highly transmissible plasmid backbone, was further disseminated to become one of the most prevalent carbapenemases in Europe, Northern Africa and the Middle East [8]. 

Although IncL/M plasmids are known for the spread of OXA-48, they have also been connected with the emergence of NDM-1 carbapenemase [9,10]. NDM-1, or New Delhi metallo-β-lactamase, is a class B1 β-lactamase first discovered in 2009 in a *K. pneumoniae* clinical isolate from a Swedish patient who had travelled to New Delhi, India [11]. Since then, it has been reported in numerous Gram-negative bacterial species and transmissible plasmids around the world, including IncN, IncA/C, IncF, IncH and IncL/M incompatibility groups [12]. In particular, a recent study performed in the UK, which analysed the sequence of 44 NDM-1-positive isolates belonging to five different bacterial species, suggested that the *bla*_NDM-1_ gene has been recently acquired on multiple occasions by IncL/M plasmids in geographically widespread isolates [13].

IncL/M plasmids were historically classified within the same incompatibility group [14]. However, these plasmids have been re-classified into IncL and IncM groups, compatible with each other and therefore belonging to two different incompatibility groups, with an overall nucleotide identity of approximately 94% [15]. IncL plasmids frequently encode OXA-48, whereas NDM-1 tend to appear in the IncM2 group [15]. One of the key differences observed between these two groups of plasmids is the inactivation of the fertility inhibition gene *tir* in IncL plasmids, which increases their conjugation rate facilitating the dissemination of the gene encoding OXA-48 [16].

In this study, a panel of clinical Enterobacterales isolates harbouring IncL or IncM2 plasmids, encoding OXA-48 and NDM-1, respectively, was phenotypically characterised using conjugation and fitness studies with the aim of evaluating the role of different bacterial hosts and plasmid backbones in the transmission potential of emergent and pandemic carbapenemase genes.

## 2. Results and Discussion

### 2.1. Uropathogenic Escherichia coli SAP1756 Harbours an IncM2 Plasmid Encoding blaNDM-1

**Figure 1:**
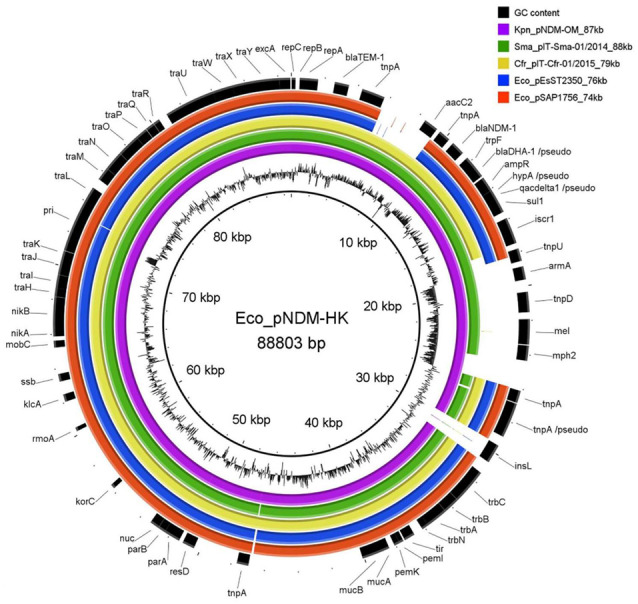
Comparison of closely related IncL/M plasmids, aligned against the reference plasmid pNDM-HK, using BLAST Ring Image Generator (BRIG) [17]. Genbank accession numbers, bacterial hosts, plasmid sizes and ring characteristics: pNDM-HK (HQ451074, *E. coli*, 88,803 bp, innermost ring, reference), pNDM-OM (JX988621, *Klebsiella pneumoniae*, 87,185 bp, purple ring), pIT-Sma-01/2014 (MH722219, *Serratia marcescens*, 88,129 bp, green ring), pIT-Cfr-01/2015 (MH722216, *Citrobacter freundii*, 78,923 bp, yellow ring), pEsST2350_SE_NDM (CP031322, *E. coli*, 75,645 bp, blue ring) and pSAP1756 ((*E. coli*, 73,743 bp, red ring). The outermost ring shows the genes identified in the reference plasmid pNDM-HK and the diagram next to the innermost ring represents the GC content of the reference plasmid.

Whole genome sequencing and genomic analysis of the uropathogenic *E. coli* SAP1756 confirmed the species was *E. coli* and belongs to ST130. Plasmid analysis identified an IncL/M conjugative plasmid carrying four AMR genes: *bla*_NDM-1_, *bla*_TEM-1_, *ble*_MBL_ and *sul1*. MOBsuite tool allowed the reconstruction of the plasmid pSAP1756 from Illumina short reads and the sequence was compared to closely related publicly available plasmids identified by BLASTn (Figure 1). 

One of the most similar plasmids in terms of size and gene content to pSAP1756 (red ring in Figure 1) found in Genbank was pEsST2350_SE_NDM (blue ring in Figure 1). Interestingly, this plasmid was found in another *E. coli* isolate of different sequence type (ST2350) collected from a human screening rectal sample, also in a South-East England hospital in 2018. A nearly identical plasmid, pKp_SE1_NDM (accession number: CM010662), was identified in *K. pneumoniae* (ST534) isolated from a human rectal sample in the same hospital in 2017 [13], suggesting that this specific plasmid variant could have been transmitting locally between different Enterobacterales species between 2017 and 2018. 

Figure 1 also shows that all the AMR genes, including *bla*_TEM-1_, *aacC2*, *bla*_NDM-1_, *bla*_DHA-1_, *sul1* and *mph2*, are clustered in the most variable region of these plasmids. The presence of genes typical in transposons and insertion elements in this variable region (e.g., *tnpA*, *tnpU*, *tnpD*, *iscr* and *insL*) reflects the ability of these plasmids to adapt to different antibiotics through the integration of mobile genetic elements carrying different AMR genes. On the other hand, plasmid genes involved in essential plasmid functions, including conjugation (e.g., *tra* genes, *trb* genes, *mobC*), replication (*rep* genes), and stability (e.g., *pemK*/*pemI* toxin-antitoxin system and *parAB* partitioning system), were grouped in the most conserved region, the plasmid backbone. 

### 2.2. IncL OXA-48-Carrying Plasmids Transfer at Higher Rates than IncM2 Harbouring NDM-1

In a previous study, clinical isolates of five different species (*E. coli*, *Enterobacter cloacae*, *Citrobacter sp.*, *K. pneumoniae* and *Klebsiella oxytoca*) isolated across the UK were found to harbour closely related IncM2 plasmids carrying *bla*_NDM-1_, suggesting inter-species horizontal gene transfer [13]. Representative isolates from that study were included here to quantify the threat posed by these emergent NDM-1 plasmids in comparison with the globally disseminated OXA-48 plasmids. 

To test their ability to transfer between common bacterial pathogens, clinical isolates from different species were used as donors in conjugation experiments, with *E. coli* MG1655 or J53 as recipients. Two subtypes of IncL/M plasmids were compared, carrying *bla*_NDM-1_ (IncM2 plasmids) or *bla*_OXA-48_ (IncL plasmids).

**Figure 2:**
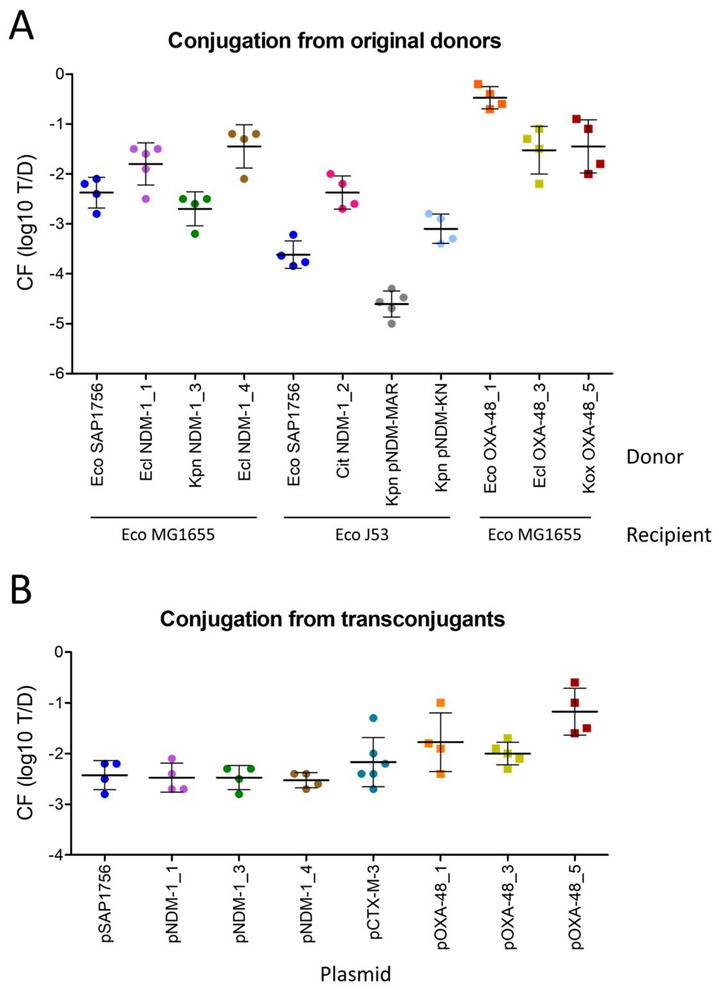
Conjugation frequency (CF) of IncL/M plasmids harbouring *bla*_NDM-1_ or *bla*_OXA-48_ genes, represented as the logarithm of transconjugants per donor obtained after 1 h conjugation at 37 °C on LB agar. (**A**) Conjugation from the original donors to *E. coli* MG1655 (rifampicin or nalidixic acid spontaneous mutants), or J53 in cases where donors were resistant to both rifampicin and nalidixic acid. Plasmids encoding NDM-1 but belonging to other incompatibility groups were added for comparison (pNDM-MAR and pNDM-KN, IncH and IncA/C, respectively). (**B**) Conjugation from the transconjugants from A in *E. coli* MG1655 (spontaneous mutant resistant to nalidixic acid) to *E. coli* MG1655 (spontaneous mutant resistant to rifampicin). Circles represent the result of individual experiments with NDM-1-carrying IncM2 plasmids (except control plasmid pCTX-M-3, which is NDM-1-negative) and squares represent CF with IncL plasmids encoding OXA-48. Horizontal lines show the mean and vertical lines the standard deviation of at least 4 independent experiments.

Conjugation of IncM2 plasmids encoding NDM-1 was successful in five out of six cases, while three out of five IncL plasmids carrying OXA-48 were able to conjugate from the original donors (Figure 2A). The donors *E. coli* NDM-1_5, *K. pneumoniae* OXA-48_2 and *Citrobacter* OXA-48_4, were unable to produce transconjugants in the tested conditions and were excluded from the results graph. The reason for this inability to transfer could potentially be related to fertility inhibition systems present in co-resident plasmids of the donor bacteria [18].

Conjugation frequencies (CF) of those plasmids able to transfer were highly diverse, ranging between 10^−4^ and 10^−1^ for IncM2 NDM-1-carrying plasmids and between 10^−2^ and 1 for IncL OXA-48 plasmids. *K. pneumoniae* pNDM-MAR and *K. pneumoniae* pNDM-KN were included as examples of alternative plasmid types carrying the *bla*_NDM-1_ gene (IncH and IncA/C, respectively), and their transfer frequencies were in the lower range compared to the IncM2 and IncL plasmids tested.

When the influence of the donor background was discarded by using *E. coli* MG1655 as the donor (Figure 2B), all IncM2 NDM-1-harbouring plasmids conjugated at homogeneous frequencies (between 10^−2^ and 10^−3^), highlighting the similarities between their plasmid backbone [13]. pCTX-M-3 plasmid was added as a control IncM2 plasmid with a different cargo (*bla*_CTX-M-3_) and its conjugation frequency was ~10^−2^. Interestingly, the three IncL OXA-48 plasmids tested in these conditions transferred at a higher rate than IncM2 plasmids, ranging between 10^−2^ and 10^−1^.

As a general rule, the IncL OXA-48-carrying plasmids are able to transfer at a slightly higher rate than IncM2 NDM-1 plasmids, a result consistent with previous studies and possibly due to the inactivation of the fertility inhibition gene *tir* in IncL plasmids [16]. However, most NDM-1 plasmids tested here were able to successfully conjugate from the original donor and the newly formed transconjugants, highlighting the importance of monitoring these emergent plasmids as potential sources of antibiotic-resistant outbreaks.

### 2.3. Most E. coli Isolates Tested as Recipients Were Not Permissive to pSAP1756 Entry

A panel of 11 pathogenic and commensal *E. coli* isolates belonging to 8 different phylogroups were selected to represent the common phylogroups and tested as recipients of pSAP1756 from the original *E. coli* donor, which belongs to phylogroup D. Some of these isolates were previously characterised in the lab by whole-genome sequencing [19] and their main features and transfer results are summarised in Table 1.

Conjugation was detected in isolates belonging to phylogroups A, B1, D and F, while no transfer was detected to those belonging to phylogroups B2, C, E and G. None of the recipients tested had resident IncL/M plasmids, so no entry exclusion was expected. Interestingly, the three B2 isolates, which are common human pathogens, were unable to receive pSAP1756, suggesting that B2 strains have mechanisms preventing the entry of these plasmids (e.g., restriction-modification, CRISPR-Cas). In future studies, it would be useful to test a higher number of sequenced recipients belonging to different phylogroups to find *E. coli* common mechanisms preventing plasmid acquisition and also to analyse the genomes of *E. coli* isolates carrying IncM2 NDM-1 plasmids to confirm the most common phylogroups carrying them as well as to understand the consequences of this potential bias.

### 2.4. pSAP1756 Is Capable of Conjugating to (and From) Different Species of Enterobacterales

*E. coli* has been used as a recipient in the previous conjugation experiments as it is one of the most important bacterial pathogens transmitting AMR genes. However, IncM2 plasmids are known to be broad-host-range and have been found in a wide range of bacterial hosts. To test their ability to transfer between different species, the studies used four type strains of Enterobacterales (belonging to the following species: *S. marcescens*, *K. pneumoniae*, *E. cloacae* and *Y. enterocolitica*) as donors and recipients of pSAP1756. Conjugation was successful in all cases, with frequencies ranging between 10^−2^ and 10^−4^ (Table 2).

### 2.5. pSAP1756 and pOXA-48_1 Transfer at Similar Rates in a Galleria Mellonella Model

**Figure 3:**
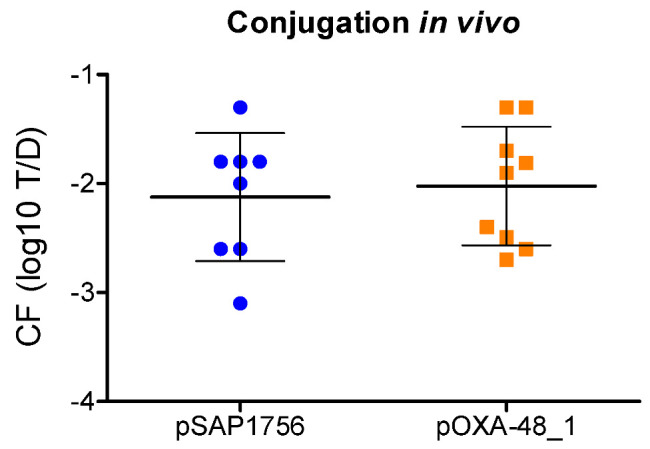
Conjugation frequency (CF) of IncM2 NDM-1-carrying plasmid pSAP1756 and IncL OXA-48-carrying plasmid pOXA48_1 using *E. coli* MG1655 resistant to nalidixic acid as donor and *E. coli* MG1655 resistant to rifampicin as recipient. Blue circles and orange squares represent the logarithm of transconjugants per donor obtained per experiment after 4 h conjugation in *Galleria mellonella* inoculated with ~10^7^ CFU of donors and recipients separately. Horizontal lines show the mean and vertical lines the standard deviation of at least 8 independent experiments.

To compare the transfer potential of plasmids harbouring NDM-1 and OXA-48 in vivo, representative examples (pSAP1756 and pOXA-48_1) were transferred to *E. coli* MG1655 and used as donors in *Galleria mellonella* conjugation experiments. These experiments were performed over a 4 h period following injection of 10^7^ CFU of donors and recipients separately into *Galleria* larvae. Conjugation frequencies observed (~10^−2^) resembled those in vitro, but with no statistically significant differences found between both plasmids (Figure 3).

### 2.6. NDM-1-Harbouring Plasmids Impose a Higher Fitness Cost for the Growth of Their Host Than Those Carrying OXA-48

**Figure 4:**
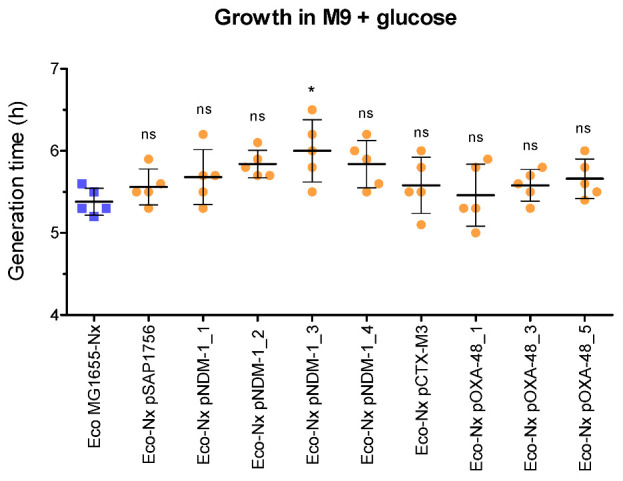
Generation time of *E. coli* MG1655 (resistant to nalidixic acid) in hours, with (orange circles) or without (blue squares) IncL/M plasmids, calculated during the exponential phase of growth in M9 minimal media supplemented with glucose. The differential growth of the host without and with plasmid represents the burden of the plasmid under these conditions. Horizontal lines show the mean and vertical lines the standard deviation of at least 5 independent experiments. One-way analysis of variance with Dunnett’s post hoc test was performed between the bacterial hosts with plasmid *versus* the control without plasmid (*: *p* < 0.05; ns: no significant difference).

In addition to horizontal gene transfer ability, fitness cost is an important parameter to test the pandemic potential of a plasmid. Growth curves were performed for *E. coli* MG1655 in the presence and absence of IncM2 and IncL plasmids carrying carbapenemase genes to estimate the burden they impose in this host. In most cases, the tested plasmids had an associated fitness cost to the host when compared to the growth rate of the same bacteria with no plasmid (Figure 4). In addition, the burden associated with OXA-48 plasmids in *E. coli* MG1655 was slightly lower than the corresponding cost of NDM-1 plasmids, which could also contribute to the increased success of OXA-48 plasmids.

## 3. Materials and Methods

### 3.1. Bacterial Cultures, DNA Extraction and Genome Sequencing

A list of the bacterial isolates used in this study can be found in Table 3. *E. coli* SAP1756 was isolated from a human urinary tract infection source in the diagnostics laboratory of a South-East England hospital in 2017 and transferred to the University of Surrey for further analysis. The isolate was aseptically cultured on MacConkey agar No3 (Oxoid, Basingstoke, UK) plates for 18–24 h at 37 °C, aerobically. A single colony was then selected for storage at −80 °C using the Microbank preservation system (Pro-Lab Diagnostics, Bromborough, UK). Bacteria stored at −80 °C were aseptically inoculated into LB broth prior to culture for 18–24 h at 37 °C, aerobically with agitation at 200 rpm. DNA was extracted using the DNeasy Blood & Tissue Kit (Qiagen, Manchester, UK) following the manufacturer’s instructions. DNA quality and quantity were confirmed using Nanodrop and DNA was shipped to the Animal and Plant Health Agency (APHA, Weybridge, UK) for whole genome sequencing using an Illumina platform.

### 3.2. Genomic Analysis

Paired-end reads were quality-checked with FastQC v0.11.9 [25] and used for genome assembly through Spades v3.15.2 [26]. The quality of the SAP1756 assembly was determined with Quast v5.0.2 [27], and Kraken v2.1.2 [28]. MLST v2.19.0 allowed the identification of the Multi-Locus Sequence Type (ST) [29,30]. ABRicate v1.0.1 in combination with NCBI AMRfinderPlus v2021-Mar-27 and PlasmidFinder v2021-Mar-27 databases were used to detect AMR genes and plasmid replicons, respectively [31,32,33]. IncL/M plasmid was reconstructed with MOBsuite v3.0.0 [34], and used as a query for NCBI BLAST Nucleotide [35]. Closely related publicly available plasmid sequences were downloaded from NCBI and compared with SAP1756 genome assembly using BLAST Ring Image Generator [17].

### 3.3. In Vitro Conjugation Assays

Donors and recipients (Table 3) were cultured on LB broth supplemented with antibiotics for 18–24 h at 37 °C, aerobically with agitation at 200 rpm. The cultures were then centrifuged for 5 min at 8000 rpm at room temperature, the supernatant was discarded, and the pellet was resuspended in sterile LB broth. OD_600_ for each culture was measured and adjusted to 4. For each individual conjugation assay, 100 µL of donor and 100 µL of recipient bacteria were mixed prior to centrifugation for 5 min at room temperature at 8000 rpm. The supernatant was discarded, the pellet resuspended in 15 µL LB broth and the final mixture of concentrated donors and recipients was spotted onto a well of a 24-well plate previously filled with 1 mL LB agar and solidified. The mixture was spread on the surface of the LB agar to create a thin layer and allowed to dry at room temperature. Conjugation was then performed for 1 h at 37 °C and stopped by resuspending the harvested mixture in 1 mL PBS 1x. Serial dilutions were prepared and 3 × 10 µL drops of each dilution were plated in LB agar with the appropriate antibiotics to select for donors, recipients and transconjugants (Table 3). Conjugation frequency was estimated by dividing the number of transconjugants (CFU/mL) by donors or recipients (CFU/mL) and the average of 3 or more independent experiments was represented in a logarithmic scale. When needed for selection purposes, spontaneous mutants of donor and recipient strains resistant to rifampicin or nalidixic acid were used, selected by growing an overnight culture of the sensitive isolate in LB agar plates supplemented with the corresponding antibiotic.

### 3.4. In Vivo Conjugation Assays

*E. coli* MG1655 resistant to nalidixic acid (carrying plasmids pSAP1756 or pOXA-48_1) and *E. coli* MG1655 resistant to rifampicin were used as donors and recipients, respectively. Donors and recipients were cultured on LB broth supplemented with antibiotics for 18–24 h at 37 °C, aerobically with agitation at 200 rpm. The cultures were centrifuged for 5 min at 8000 rpm at room temperature, the supernatant was discarded, and the pellet was resuspended in sterile PBS 1x. A volume of 10 µL of donors and 10 µL of recipients containing ~10^7^ CFU were separately injected into the right and left prolegs of *Galleria mellonella* larvae (TruLarv, Biosystems Technology, Crediton, UK) using microliter syringes (Hamilton, Bonaduz, Switzerland). Conjugation was permitted for 4 h at 37 °C. Larvae were euthanised by decapitation and then homogenised in 200 µL of sterile PBS 1x with a Micro Tissue Homogenizer (BioMasherII, Fisher Scientific, Loughborough, UK). As previously described, serial dilutions were prepared and 3 x 10 µL drops of each dilution were plated in LB agar with the appropriate antibiotics to select for donors (meropenem 0.25 μg/mL), recipients (rifampicin 50 μg/mL) and transconjugants (rifampicin 50 μg/mL + meropenem 0.25 μg/mL). Conjugation frequency was estimated by dividing transconjugants (CFU/mL) by donors (CFU/mL). The results represented the average of at least 8 independent experiments on a logarithmic scale. 

### 3.5. Fitness Cost Experiments

Bacterial isolates were cultured on LB broth supplemented with the appropriate antibiotics for 18–24 h at 37 °C, aerobically and with agitation at 200 rpm. Cultures were centrifuged for 5 min at 8000 rpm at room temperature and resuspended in M9 minimal media supplemented with 0.4% glucose, adjusting to an OD_600_ of 0.01 or 0.001. A volume of 100 µL of diluted cultures was added to 96-well microplates and incubated for 24 h at 37 °C, aerobically in a Spark 10M microplate reader. OD_600_ was measured every 10 min after 20 s of double orbital shaking at 1 mm of amplitude and 270 rpm of frequency. The absorbance results (Ln2) were plotted over time and the generation time was estimated from the exponential phase of the graph by dividing Ln2 by the slope of the linear trendline. At least 5 growth curves were used to calculate the average generation time of a given isolate. One-way analysis of variance (ANOVA) with Dunnett’s post hoc test comparing each plasmid-carrying bacterial host *versus* the control (host without plasmid) was performed using GraphPad Prism. 

## 4. Conclusions

The studies reported here demonstrated that IncL/M plasmids are vehicles of *bla*_NDM-1_ and *bla*_OXA-48_ genes able to disseminate these carbapenemase genes to a broad number of bacterial hosts. In general, the emergent IncM2 NDM-1-harbouring plasmids tested under the conditions of this study had a lower transfer rate and higher fitness cost when compared with the pandemic IncL OXA-48-carrying plasmids, a potential cause for the more extensive global spread of OXA-48 plasmids. However, it is important to continue monitoring plasmids carrying carbapenemase and other resistance genes that could be a threat to human and animal health due to the rapid evolution and adaptability of mobile genetic elements.

## Figures and Tables

**Table 1:** Pathogenic and commensal *E. coli* isolates used as recipients in conjugation experiments.

*E. coli* Recipient	Type	Phylogroup	Source	Year	Country	CF (log10 T/R)
MG1655 (RifR)	Commensal	A	Human	1922	US	−2.1 ± 0.2
SAP2531 (RifR)	Pathogenic	A	Human	2018	UK	−3.8 ± 0.7
SAP2592 (RifR)	Pathogenic	B1	Human	2018	UK	−3.4 ± 0.6
NCTC 12241 (RifR)	Pathogenic	B2	Human	1946	US	N.D.
NCTC 13441 (NxR)	Pathogenic	B2	Human	2003	UK	N.D.
SAP2492 (RifR)	Pathogenic	B2	Human	2018	UK	N.D.
SAP2188 (NxR)	Pathogenic	C	Avian	2018	UK	N.D.
SAP2526 (NxR)	Pathogenic	D	Human	2018	UK	−4.4 ± 0.4
SAP2573 (RifR)	Pathogenic	E	Human	2018	UK	N.D.
SAP2485 (RifR)	Pathogenic	F	Human	2018	UK	−3.6 ± 0.2
SAP2180 (RifR)	Pathogenic	G	Avian	2018	UK	N.D.

CF: conjugation frequency (mean ± standard deviation) of IncM2 plasmid pSAP1756 using the original host as donor (SAP1756, phylogroup D). N.D.: no transconjugants were detected in one or more experiments. RifR: resistant to rifampicin. NxR: resistant to nalidixic acid. T: transconjugants/mL. R: recipients/mL.

**Table 2:** Conjugation frequency of IncM2 plasmid pSAP1756 from the original host to different Enterobacterales species and from these different species to *E. coli* MG1655.

**Donor**	**Plasmid**	**Recipient**	**CF (log10 T/R)**
*E. coli* SAP1756	pSAP1756	*E. coli* MG1655 (RifR)	−2.1 ± 0.2
*E. coli* SAP1756	pSAP1756	*S. marcescens* NCTC 13382 (RifR)	−3.0 ± 0.2
*E. coli* SAP1756	pSAP1756	*K. pneumoniae* NCTC 9633 (RifR)	−3.6 ± 0.4
*E. coli* SAP1756	pSAP1756	*E. cloacae* NCTC 13380 (RifR)	−4.0 ± 0.1
*E. coli* SAP1756	pSAP1756	*Y. enterocolitica* NCTC 12982 (RifR)	−2.4 ± 0.3
**Donor**	**Plasmid**	**Recipient**	**CF (log10 T/D)**
*E. coli* MG1655 (RifR)	pSAP1756	*E. coli* MG1655 (NxR)	−2.4 ± 0.2
*S. marcescens* NCTC 13382 (RifR)	pSAP1756	*E. coli* MG1655 (NxR)	−2.3 ± 0.4
*K. pneumoniae* NCTC 9633 (RifR)	pSAP1756	*E. coli* MG1655 (NxR)	−2.5 ± 0.7
*E. cloacae* NCTC 13380 (RifR)	pSAP1756	*E. coli* MG1655 (NxR)	−1.9 ± 0.2
*Y. enterocolitica* NCTC 12982 (RifR)	pSAP1756	*E. coli* MG1655 (NxR)	−1.8 ± 0.5

CF: conjugation frequency (mean ± standard deviation); RifR: resistant to rifampicin; NxR: resistant to nalidixic acid; T: transconjugants/mL; D: donors/mL; R: recipients/mL.

**Table 3:** Bacterial strains and plasmids used in this study.

Species	Isolate	Plasmid	Inc	AMR Gene	D/R	Selection	Reference
*E. coli*	SAP1756	pSAP1756	IncM2	*bla*_NDM-1_	D	Mer_0.25_	This work
*E. coli*	SAP2180				R	Nx_50_	This work
*E. coli*	SAP2188				R	Nx_50_	This work
*E. coli*	SAP2485				R	Rif_50_	This work
*E. coli*	SAP2492				R	Rif_50_	[19]
*E. coli*	SAP2526				R	Nx_50_	[19]
*E. coli*	SAP2531				R	Rif_50_	[19]
*E. coli*	SAP2573				R	Rif_50_	[19]
*E. coli*	SAP2592				R	Nx_50_	[19]
*E. cloacae*	NDM-1_1	pNDM-1_1	IncM2	*bla*_NDM-1_	D	Mer_0.25_	[13]
*Citrobacter*	NDM-1_2	pNDM-1_2	IncM2	*bla*_NDM-1_	D	Mer_0.25_	[13]
*K. pneumoniae*	NDM-1_3	pNDM-1_3	IncM2	*bla*_NDM-1_	D	Mer_0.25_	[13]
*E. cloacae*	NDM-1_4	pNDM-1_4	IncM2	*bla*_NDM-1_	D	Mer_0.25_	[13]
*E. coli*	NDM-1_5	pNDM-1_5	IncM2	*bla*_NDM-1_	D	Mer_0.25_	[13]
*E. coli*	OXA-48_1	pOXA-48_1	IncL	*bla*_OXA-48_	D	Mer_0.25_	This work
*K. pneumoniae*	OXA-48_2	pOXA-48_2	IncL	*bla*_OXA-48_	D	Mer_0.25_	This work
*E. cloacae*	OXA-48_3	pOXA-48_3	IncL	*bla*_OXA-48_	D	Mer_0.25_	This work
*Citrobacter*	OXA-48_4	pOXA-48_4	IncL	*bla*_OXA-48_	D	Mer_0.25_	This work
*K. oxytoca*	OXA-48_5	pOXA-48_5	IncL	*bla*_OXA-48_	D	Mer_0.25_	This work
*E. coli*	MG1655	pCTX-M-3	IncM2	*bla*_CTX-M-3_	D	Ctx_2_	[20]
*K. pneumoniae*	TCKpnC	pNDM-MAR	IncH	*bla*_NDM-1_	D	Mer_0.25_	[21]
*K. pneumoniae*	Kp7	pNDM-KN	IncA/C	*bla*_NDM-1_	D	Ctx_2_	[22]
*E. coli*	MG1655				R	Nx_50_/Rif_50_	[23]
*E. coli*	J53				R	NaN_3100_	[24]
*S. marcescens*	NCTC 13382				R	Rif_50_	
*K. pneumoniae*	NCTC 9633				R	Rif_50_	
*E. cloacae*	NCTC 13380				R	Rif_50_	
*Y. enterocolitica*	NCTC 12982				R	Rif_50_	
*E. coli*	NCTC 12241				R	Rif_50_	
*E. coli*	NCTC 13441				R	Nx_50_	

Inc: incompatibility group; D: donor; R: recipient; Mer_0.25_: meropenem 0.25 μg/mL; Nx_50_: nalidixic acid 50 μg/mL; Rif_50_: rifampicin 50 μg/mL; Ctx_2_: cefotaxime 2 μg/mL; NaN_3100_: sodium azide 100 μg/mL).

## Data Availability

The data for this study have been deposited in the European Nucleotide Archive (ENA) at EMBL-EBI under accession number PRJEB57396 (https://www.ebi.ac.uk/ena/browser/view/PRJEB57396, accessed on 16 November 2022).
